# Creasote in Menorrhagia

**Published:** 1853-04

**Authors:** John W. Crooks

**Affiliations:** Rockport, Ind.


### THE WESTERN JOURNAL OF MEDICINE AND SURGERY.

APRIL, 1853.

### Article 1.—*Creasote in Menorrhagia.* By Dr. John W. Crooks, of Rockport, Ind.

Creasote has been used or recommended in almost every variety of hemorrhage, but the writer does not remember to have seen any notice of it as an internal remedy in menorrhagia. Having employed it in several of the complaints for which it is generally recommended, and found it efficient and useful, he was induced to make trial of it in this troublesome affection.

The first case in which he resorted to it was one of a most obstinate character. The patient, about thirty-five years old, had been once married, but for five or six years was a widow, and all that time was a great sufferer from menorrhagia. The hemorrhage usually occurred at the regular menstrual period, and continued from ten to fourteen days. The effect of this drain upon her health, as can readily be imagined, was most disastrous. There was an extremely emaciated, feeble, and anemic condition of her system.

The case had resisted the treatment of several physicians of experience and character. The writer prescribed creasote, and the effects were satisfactory beyond any thing that he could have expected, or even ventured to hope. In the first four weeks it was evident that improvement had begun ; in six weeks she was decidedly convalescent ; and in two months she regarded herself as comparatively well. She took no other medicine during that time. A slight return of hemorrhage was experienced about this time, the patient having discontinued the use of the creasote. She was then ordered to take it for ten or twelve days before the menstrual periods ; and now, after the lapse of more than two years, she remains free from the complaint.

Since this case was treated, the writer has had an opportunity, with his friend and partner, Dr. DeBruler, of administering the creasote in ten or a dozen similar cases, and with as gratifying results. We have always found the remedy effectual, and have not had to employ any other styptic The form in which we prescribe it is the following :

℞.—Creasote, ʒj.

Alcohol, ʒij.

Spts. Lavend comp., ʒiij.

Aq. destillat., ʒx.—M.

Of this, give from half a drachm to one drachm every four hours, until the hemorrhage is partially or wholly arrested. After the paroxysm is over, it may be continued during the menstrual intervals, two or three times daily for several weeks, or even months, if the case should require it. For the nervous irritability often attending these cases, it will be found occasionally beneficial to combine an opiate with the creasote.

This article is recommended with great confidence in the complaint under notice, and if, in the hands of other practitioners, it should prove as efficient as it has done in the writer's, it must soon rank high among the remedies in such cases.

ROCKPORT, Ind., February 25, 1853.

